# Nutrient deprivation and lysosomal stress induce activation of TFEB in retinal pigment epithelial cells

**DOI:** 10.1186/s11658-019-0159-8

**Published:** 2019-05-27

**Authors:** Hsuan-Yeh Pan, Abdulla H. Alamri, Mallika Valapala

**Affiliations:** 1 School of Optometry, Indiana University, Bloomington, IN 47405 USA; 2 State University of New York College of Optometry, 33 42nd St., New York, NY 10036 USA

**Keywords:** Nutrient deprivation, Lysosomal stress, Autophagy

## Abstract

**Background:**

Induction of lysosomal function and autophagy is regarded as an adaptive mechanism in response to cellular stress. The transcription factor EB (TFEB) has been identified as a master regulator of lysosomal function and autophagy. TFEB is a member of the microphthalmia family of bHLH-LZ transcription factors that includes other members such as micropthalmia-associated transcription factor (MITF), TFE3, and TFEC. TFEB controls lysosome biogenesis and autophagy by upregulation of a family of genes belonging to the Coordinated Lysosomal Expression and Regulation (CLEAR) network. Here, we investigated the expression of TFEB in cells subjected to nutrient deprivation and lysosomal stress. We studied transcriptional induction of TFEB-regulated genes in response to nutrient deprivation and lysosomal stress in retinal pigment epithelial (RPE) cells. Furthermore, we also investigated the induction of autophagy and lysosomal genes upon overexpression of constitutively active form of TFEB.

**Methods:**

Expression of TFEB and MITF protein levels were evaluated in cells subjected to prolonged periods of nutrient deprivation. mRNA levels of the CLEAR network genes was measured by quantitative real time PCR (qRT-PCR) analysis in cells deprived of nutrients, treated with ammonium chloride and upon overexpression of constitutively active TFEB. Immunostaining with LC3 antibody was used to measure autophagy flux. Labeling with lysoTracker dye was used to assess lysosomes.

**Results:**

Our results show that nutrient deprivation increases protein levels of TFEB and MITF in ARPE-19 cells. Nutrient stress induces the expression of lysosomal (LAMP1, CTSD MCOLN1, SGSH) and autophagy (BECN1) genes. Lysosomal stress also increases the expression of lysosomal (ATP6V0A1 and LAMP1) and autophagy (p62 and BECN1) genes. Our results show that overexpression of constitutively active TFEB also induces the expression of CLEAR network genes.

**Conclusions:**

Collectively, these observations suggest that nutrient stress induces the protein expression of both MITF and TFEB in ARPE-19 cells. TFEB-regulated transcriptional program plays an important role in adaptive response of cells during both nutrient and lysosomal stress.

## Introduction

The retinal pigment epithelium (RPE) serves many physiological roles responsible for the maintenance of homeostasis in the retina [1]. One of the functions of the RPE is phagocytosis and degradation of the shed photoreceptor outer segments, which is important for photoreceptor renewal and maintenance. RPE cells are post- mitotic and the amount of material processed by these cells in their lifetime is higher than any other cell type in the body [2]. Phagocytosis is a complex process mediated by several steps, including recognition of the photoreceptor outer segments (POS), binding, internalization, formation of a phagosome and finally degradation [3]. Phagosomes containing internalized photoreceptor outer segments fuse with acidic lysosomes in the RPE for subsequent degradation [4]. Because of the post mitotic nature of the RPE cells, impaired degradation and clearance of the phagocytosed outer segments results in the buildup of undigested or partially digested cellular material in the RPE. Lysosomes, which are the terminal organelles involved in processing of the phagosomes decline in function with age [5]. Accumulation of lipofuscin also inhibits degradation of phagosomes and thereby contributing to accumulation of cellular material in the RPE [6]. In addition to phagocytosis, autophagy, a process involved in the processing of the cellular components is also active in the RPE. The process of autophagy begins with the sequestration of cellular components like senescent organelles and damaged proteins into a double membrane organelle called the autophagosome [7]. In a manner that is similar to the phagosome, autophagosomes fuse with the lysosomes for degradation [8, 9]. Since both phagocytosis and autophagy processes require lysosomes for their completion, impaired lysosomal function can significantly affect these processes and cause accumulation of cellular material in the RPE [10, 11]. Hence, strategies that can induce the degradative ability of the lysosomes can have a positive effect on enhancing cellular clearance in the RPE.

A wide variety of genes are involved in lysosomal biogenesis, transport and maturation and are important for the maintenance of lysosomal function [12]. The Coordinated Lysosomal Expression and Regulation (CLEAR) network comprises several genes associated with lysosomal biogenesis, lysosomal acidification and autophagy pathway [13]. Under basal conditions of adequate nutrient availability, transcription factor EB (TFEB) is predominantly cytosolic and maintained in an ‘off’ state. During cellular stress, TFEB is released from its cytosolic sequestration and translocates to the nucleus to facilitate the expression of genes in the CLEAR network [13]. TFEB is also known to positively regulate its expression under conditions of nutrient deprivation [14].

Previous studies have suggested that TFEB is negatively regulated by the mechanistic target of Rapamycin complex 1 (mTORC1) by phosphorylation and cytosolic retention [15, 16]. It is previously known that phosphorylation of TFEB at two residues, S142 and S211, influences its nuclear localization and activity [15, 16]. Under conditions of adequate nutrient supply, dephosphorylation of TFEB promotes its nuclear translocation and activation of CLEAR network genes. TFEB is also known to be dephosphorylated by a phosphatase calcineurin, which in turn is regulated by cellular levels of Ca^2+^ [17]. Previous studies have suggested that a TFEB-regulated transcriptional program induces flux through lysosomal degradative pathways and prevents substrate accumulation in several neurodegenerative diseases [18, 19]. Induction of the TFEB-regulated transcriptional program is an adaptive mechanism in response to a variety of cellular stressors [20]. In addition to TFEB, nuclear translocation of other members of the microphthalmia family of bHLH-LZ transcription factors (MiT/TFE), MITF and TFE3 are known to constitutively activate autophagy and lysosomal pathway in several cancer cells [21]. In this study, we investigated the effect of cellular stressors on the induction of TFEB and CLEAR network genes in retinal pigment epithelial cells. Our results show that genes associated with maintenance of lysosomal function and autophagy are induced as an adaptive mechanism in the RPE in response to cellular stressors like nutrient deprivation and lysosomal stress.

## Methods

### Antibodies

The following antibodies were used in this study. TFEB (A303-673A-T, Bethyl Laboratories), MITF (ab140606, abcam), LC3 (PM036, MBL International), SQSTM1/P62 (ab109012, abcam).

### Cell culture and animal studies

Adult Retinal Pigment Epithelial cell line-19 (ARPE-19) cells were cultured in DMEM/F12 with L-Glutamine and 15 mM HEPES (Gibco, Thermo Fisher Scientific) along with 10% Fetal Bovine Serum (Hyclone, GE Healthcare Life Sciences) and 1% Antibiotic-Antimicotic (Gibco, Thermo Fisher Scientific). For starvation, the cells were cultured in Earle’s Balanced Salt Solution with calcium and magnesium for 24–72 h. C57BL/6 J mice were withheld food for 24–72 h and the mice were provided with water during this period. The experimental procedures were approved by the Institutional Animal Care and Use Committee, Indiana University/School of Optometry and conformed to the ARVO Statement for the Use of Animals in Ophthalmologic and Vision Research.

### Plasmid and transfection

pEGFP-N1-TFEB was a gift from Shawn Ferguson (Addgene plasmid # 38119) [15]. S142A and S211A mutations were introduced by site-directed mutagenesis (GenScript Corporation, Piscatway, NJ, USA). Lipofectamine 2000 (Invitrogen) was used as a transfection reagent for plasmid transfection.

### Labeling of lysosomes

ARPE-19 cells were seeded on 8 chamber slides and treated with EBSS (Gibco, Thermo Fisher Scientific) for 24 h. LysoTracker Green DND-26 (Invitrogen, Thermo Fisher Scientific) was to assay lysosomal number. After nutrient deprivation, the cell medium was replaced by pre-warmed (37 °C) probe-containing medium and incubated for 2 h at 37 °C. The cells were mounted by Prolong Gold antifade reagent with DAPI (Life technology), examined by Zeiss microscope equipped with a camera (ApoTome.2; Carl Zeiss).

### Immunostaining and microscopy

ARPE-19 cells were seeded on 8-well chamber slides and subjected to nutrient deprivation for 48 h. After treatment, cells were fixed by 4% paraformaldehyde and permeabilized with 0.5% Triton X-100 diluted in PBS. The cells were treated with blocking buffer (5% BSA and 0.5% Tween-20 in 1× PBS) containing 10% goat serum (MP biomedicals.) LC3 (MBL International) antibodies were added and incubated overnight at 4 °C. Secondary antibody was added the following day and incubated for 1 h at room temperature. The slides were mounted by Prolong Gold antifade reagent with DAPI (Life technology) and imaged by Zeiss microscope (ApoTome.2; Carl Zeiss).

### Immunoblotting

ARPE-19 cells were seeded in 6-wells plates and then treated with EBSS (Gibco) for 24–72 h. Total cell lysates and RPE choroid extracts were subjected to immunoblotting with TFEB (1:1000) and MITF (1:200) antibodies.

### Quantitative real time-PCR

RNA was isolated from ARPE-19 cells by RNeasy Mini Kit (QIAGEN). 400 ng of RNA was converted to cDNA by RNA-to-cDNA Kit (Applied Biosystems). Real-time PCR was performed using SsoAdvanced™ SYBR® Green Supermix (Bio-Rad). mRNA expression was analyzed for the following genes using appropriate primers: Beclin 1 (BECN1), Lysosomal-associated membrane protein 1 (LAMP1), Cathepsin D (CTSD), Sequestosome 1/p62, N-sulfoglucosamine sulfohydrolase (SGSH), Mucolipin 1 (MCOLN1), ATPase H+ Transporting V0 Subunit A1 (ATP6V0A1), Microtubule-associated proteins 1A/1B light chain 3B (MAP1LC3B).

### Statistical analysis

All data are presented as mean ± standard deviation. Two-tailed t test was used for analysis. * *p*-value <0.05, ** *p*-value <0.01 is considered statistically significant.

## Results

### Induction of TFEB and associated transcriptional program in cells subjected to nutrient deprivation

**Fig. 1:**
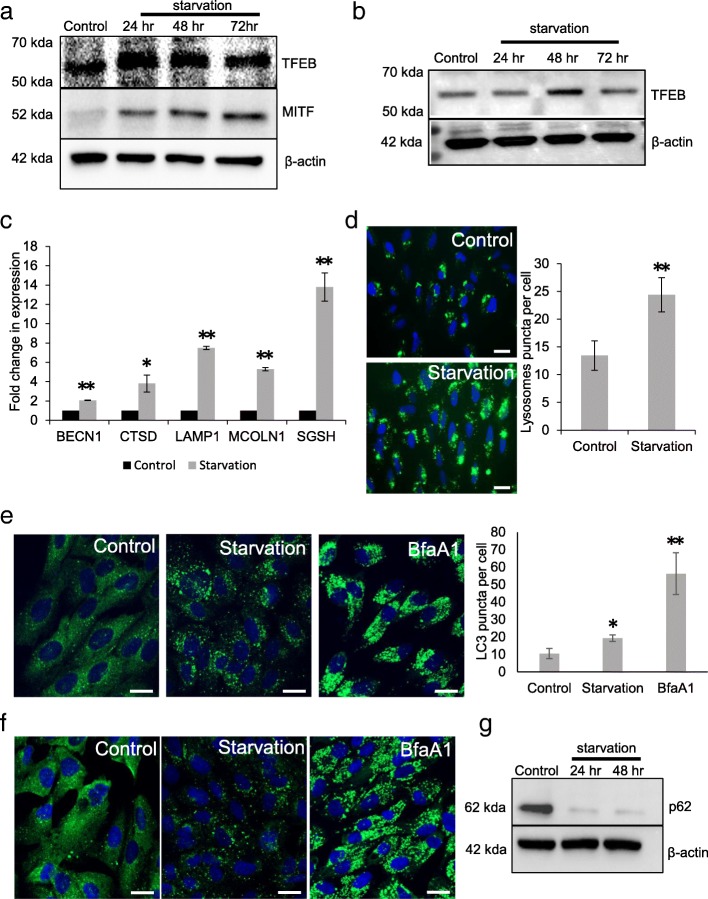
Induction of TFEB and CLEAR network genes in cells subjected to starvation. **a** ARPE-19 cells were subjected to 24–72 h period of nutrient deprivation and the expression levels of TFEB and MITF was measured by immunoblotting. **b** Expression of TFEB in RPE choroid extracts from mice subjected to 24–72 h period of nutrient withdrawal. **c** Quantitative real time PCR (qRT-PCR) analysis was performed to analyze the expression of autophagy and lysosomal genes: BECN1, CTSD, LAMP1, MCOLN1 and SGSH in ARPE-19 cells subjected to nutrient deprivation for 48 h. **d** LysoTracker staining of ARPE-19 cells in cells subjected to nutrient deprivation for 24 h. **e** ARPE-19 cells were subjected to a 24 h period of nutrient deprivation to determine the cellular expression of LC3 by immunostaining. **f** Immunostaining with p62 antibody to determine cellular levels of p62 in cells subjected to nutrient deprivation and Bafilomycin treatment for 24 h. **g** Immunoblot analysis to determine the expression of p62 in cells subjected to nutrient deprivation for 24 and 48 h. Values represent mean ± s.d. of three independent experiments. For animal experiments *n* = 3 mice were used per group. Student’s t-test (two tailed) was used. For quantification of images Mann–Whitney U test was used. *P-value <0.05 and **P-value <0.01. Scale = 20 μm

Here, we investigated the expression of MITF, TFEB and mRNA levels of TFEB-regulated CLEAR network genes upon nutrient deprivation. Immunoblotting with TFEB antibody revealed increased cellular expression of TFEB in ARPE-19 cells subjected to prolonged periods of nutrient deprivation (24–72 h) (Fig. 1a). Our results show that the expression of TFEB was significantly induced upon nutrient deprivation for 24 and 48 h respectively compared to control cells. We also observed an induction in the mRNA levels of TFEB in cells deprived of nutrients (data not shown). In addition to TFEB, we also investigated the expression of Microphthalmia-associated transcription factor (MITF), an important transcription factor known to play key roles in differentiation of RPE [22]. MITF and TFEB are members of microphthalmia family of transcription factors (MiT). Recent evidence suggests that MITF family of transcription factors are involved in regulating lysosomal biogenesis by upregulating the transcription of several lysosomal genes [23]. Our results show that in cells subjected to starvation for prolonged periods of time (24–72 h) elevated expression of MITF was observed compared to the cells cultured with serum and amino acid supplemented media. We observed a significant increase in the expression of MITF in cells subjected to starvation for 24, 48 and 72 h respectively compared to control cells (Fig. 1a). We also investigated the levels of TFEB in the RPE-choroid extracts from mice subjected to a 24–72 h period of nutrient deprivation. Our results show an increase in the protein levels of TFEB in mice subjected to starvation stress compared to control mice (Fig. 1b). We also investigated the expression of a few critical genes important for lysosomal function and autophagy. Cathepsin D is expressed in the RPE and is important for the degradation of photoreceptor outer segments [24]. Our results (Fig. 1c) show that nutrient deprivation increases the transcription of cathepsin D in the RPE. LAMP-1 is responsible for the maintenance of lysosomal structural and functional integrity [25]. The expression of LAMP-1 was significantly induced in cells subjected to nutrient deprivation. We also observed an increase in the expression of MCOLN1, BECN1 and SGSH upon starvation (Fig. 1c). Next, we investigated whether lysosomes were induced upon nutrient deprivation. The cells were loaded with LysoTracker dye to label the lysosomes. LysoTracker dyes are easily cell permeable and are retained in acidic organelles, allowing an assessment of lysosomal acidity. Cells subjected to 24 h of nutrient deprivation showed a significant increase in lysoTracker-staining compared to control cells (Fig. 1d). Next, we investigated the induction of autophagy upon nutrient deprivation. Immunostaining with LC3 antibody revealed a significant induction of LC3 puncta in cells subjected to nutrient deprivation compared to control cells. In cells cultured under normal conditions, LC3 was observed to be evenly distributed in the cytosol. In cells subjected to starvation, however, LC3 staining was observed to be punctate and localized in the perinuclear region. Quantification of LC3 puncta using ImageJ software showed a significant increase in LC3 puncta in starved cells compared to control cells. Accumulated LC3 puncta was observed in cells treated with the lysosomal disrupting agent, Bafilomycin A1 (Fig. 1e). We also investigated the expression of p62, also called Sequestosome-1, which is involved in targeting substrates to the autophagy pathway. Immunostaining studies indicated a distribution of p62-positive puncta around the nucleus in cells subjected to nutrient deprivation for 24 h compared to cells cultured under normal conditions. Intense accumulation of P62 puncta was observed in cells treated with the lysosomal inhibitor, Bafilomycin A1 (Fig. 1f). Immunoblot analysis revealed a decrease in p62 levels in cells subjected to nutrient deprivation (Fig. 1g).

### Transcriptional induction of TFEB and CLEAR network genes in cells subjected to treatment with ammonium chloride

**Fig. 2:**
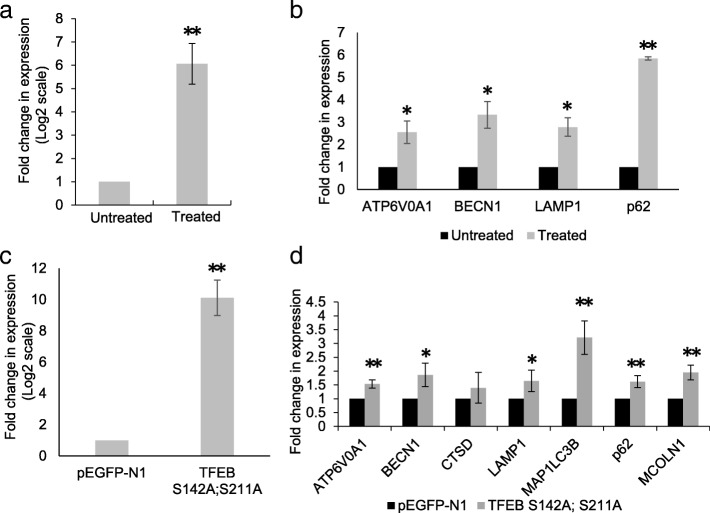
Transcriptional induction of TFEB and CLEAR network genes in cells subjected to treatment with ammonium chloride and upon overexpression of TFEB. **a** Expression levels of TFEB transcripts in ARPE-19 cells treated with ammonium chloride. **b** The expression of autophagy and lysosomal genes: ATP6V0A1, BECN1, LAMP1 and p62was analyzed by qRT-PCR in cells treated with ammonium chloride. **c** ARPE-19 cells were transfected with a constitutively active mutant of TFEB (S142A; S211A) and the cellular levels of TFEB transcripts was analyzed by qRT-PCR. **d** qRT-PCR analysis of the expression of lysosomal (LAMP-1, CTSD, MCOLN1 and ATP6V0A1) and autophagy genes (BECN1, MAP1LC3B and p62) upon overexpression of constitutively active TFEB in ARPE-19 cells. Values represent mean ± s.d. of three independent experiments. Student’s t-test (two tailed) was used for analysis *P-value <0.05; **P-value <0.01

Lysosomes play a crucial role in the maintenance of cellular homeostasis and disruption of lysosomal function results in impaired clearance of cellular material [8]. We tested whether TFEB-regulated transcriptional network is induced in cells with lysosomal impairment. We investigated the effect of TFEB and its downstream targets upon disrupting lysosomal function by treatment with ammonium chloride. qRT-PCR analysis revealed that treatment with ammonium chloride (5 mM for 72 h) induced the expression of TFEB (Fig. 2a) followed by a concomitant induction in the expression of both autophagy (BECN1 and p62) and lysosomal genes (LAMP1 and ATP6V0A1) in the CLEAR network (Fig. 2b). In addition, we investigated if overexpression of a constitutively active mutant of TFEB (S142A; S211A) induces autophagy and lysosomal genes in the RPE. Transfection of ARPE-19 cells with TFEB constitutively active mutant, TFEB S142A; S211A showed an increase in the expression of TFEB transcripts compared to cells transfected with vector control (Fig. 2c) followed by a concomitant induction in the expression of TFEB-regulated lysosomal (ATP6V0A1, MCOLN1, CTSD, LAMP1) and autophagy (BECN1, p62, MAP1LC3B) genes compared to vector control transfected cells (Fig. 2d).

## Discussion

In the present study, we investigated the role of TFEB-regulated genes during cellular response to nutrient deprivation and lysosomal stress. Our results show that the transcription of TFEB-regulated lysosomal and autophagy genes is significantly induced when cells were deprived of nutrients for a prolonged periods of time and subjected to lysosomal stress. In addition to an induction of TFEB-regulated genes, we also observed an increase in the endogenous expression of both TFEB and MITF during prolonged periods of starvation in both ARPE-19 cells and RPE-choroid extracts. Furthermore, overexpression of constitutively active TFEB induces transcription of CLEAR network genes in ARPE-19 cells.

Recent studies have established that lysosomes play a major role in sensing nutrient status of the cell and thereby coordinating cellular processes [26, 27]. Under conditions of nutrient deprivation, lysosomes process cellular material from the autophagy pathway, and induction of lysosomal function in these conditions facilitates efficient clearance of autophagy substrates [8]. Induction of autophagy also functions as a protective mechanism in response to nutrient deprivation [8]. Co-ordinated induction of lysosomal function and autophagy pathway facilitates cell survival under stress. Previous studies have shown that starvation triggers the release of lysosomal Ca^2+^, which activates calcinuerin, a Ca^2+^-dependent phosphatase that dephosphorylates and promotes nuclear translocation of TFEB [17]. MCOLN1 is a major Ca^2+^ channel in the lysosomes that is responsible for the release of Ca^2+^ under conditions of nutrient deprivation [28]. Our data shows that prolonged exposure to nutrient deprivation results in transcriptional induction of MCOLN1 in the RPE.

Under conditions of nutrient stress, TFEB translocates to the nucleus to facilitate coordinated induction of lysosomal and autophagy genes in the CLEAR network [29]. In the present study, we show transcriptional induction of TFEB-regulated genes belonging to lysosomal and autophagy pathway in response to nutrient deprivation. Interestingly, our work also shows that inhibition of lysosomal function in response to treatment with ammonium chloride induces the expression of TFEB and some of the TFEB-regulated genes. Ammonium chloride, a widely accepted lysosomotropic agent accumulates in the lysosomes in a protonated form and increases lysosomal pH [30]. Studies have also suggested that ammonium chloride is known to inhibit the fusion of phagosomes with lysosomes and is also known to directly affect the completion of the autophagy pathway [31]. Previous studies have also shown activation of TFEB in the presence of lysosomal stress caused by lysosomotropic agents [32, 33]. Taken together, our results suggest that both nutrient deprivation and lysosomal stress induces TFEB-regulation transcriptional network in the RPE.

## Conclusion

Our results also show that nutrient deprivation induces protein levels of TFEB and MITF in RPE cells, suggesting that TFEB auto-regulates its own expression in conditions of cellular stress. Transcriptional activation of a few TFEB-regulated CLEAR network genes was observed in cells subjected to nutrient deprivation and also in cells treated with ammonium chloride. Overexpression of constitutively active form of TFEB also induces some of the TFEB-regulated CLEAR network genes. In conclusion, our results provide evidence supporting the role of TFEB as an important regulator of cellular homeostasis in response to nutrient deprivation and lysosomal stress.

## Abbreviations

ARPE-19: Adult Retinal Pigment Epithelial cell line-19
ATP6V0A1: ATPase H+ Transporting V0 Subunit A1
BECN1: Beclin 1
CLEAR: Coordinated Lysosomal Expression and Regulation
CTSD: Cathepsin D
LAMP1: Lysosomal-associated membrane protein 1
LC3: Microtubule-associated proteins 1A/1B light chain 3B
MCOLN1: Mucolipin 1
MITF: Microphthalmia-Associated Transcription Factor
mTORC1: mechanistic target of Rapamycin complex 1
RPE: Retinal pigment epithelium
SGSH: N-sulfoglucosamine sulfohydrolase
SQSTM1: Sequestosome 1
TFEB: Transcription factor EB
